# The usefulness of a complete blood count in the prediction of the first episode of schizophrenia diagnosis and its relationship with oxidative stress

**DOI:** 10.1371/journal.pone.0292756

**Published:** 2023-10-12

**Authors:** Dariusz Juchnowicz, Michał Dzikowski, Joanna Rog, Napoleon Waszkiewicz, Kaja Hanna Karakuła, Anna Zalewska, Mateusz Maciejczyk, Hanna Karakula-Juchnowicz

**Affiliations:** 1 Department of Psychiatric Nursing, Medical University of Lublin, Lublin, Poland; 2 1st Department of Psychiatry, Psychotherapy and Early Intervention, Medical University of Lublin, Lublin, Poland; 3 Laboratory of Human Metabolism Research, Department of Dietetics, Warsaw University of Life Sciences, Warsaw, Poland; 4 Department of Psychiatry, Medical University of Bialystok, Choroszcz, Poland; 5 Department of Restorative Dentistry and Experimental Dentistry Laboratory, Medical University of Bialystok, Bialystok, Poland; 6 Department of Hygiene, Epidemiology and Ergonomics, Medical University of Bialystok, Bialystok, Poland; Oregon State University, UNITED STATES

## Abstract

A complete blood count (CBC) is a routinely performed blood examination. Only a few studies assess the relationship between CBC and oxidative stress (OS) in schizophrenia (SZ). The aim of the study was to assess the utility of CBC in the prediction of SZ diagnosis, and the relationship between CBC and OS. The study included: 47 individuals with the first episode of psychosis (26 drug-naive: FEP-nt; 21 patients under antipsychotic treatment: FEP-t) and 30 healthy persons (control group, HC). CBC and oxidative stress-related parameters were assessed in blood samples. The FEP group had higher levels of WBC, MCHC, NEU, MONO, EOZ, BASO, and %EOZ compared to HC (p<0.05). Various relationships between OS and CBC were found, and this connection was significantly different between healthy individuals and patients. The most promising C&RT model for discriminating FEP from HC was combining monocytes, eosinophils, and neutrophils (accuracy: 77%, **95%CI = 0.67–0.87**). The analysis singled out WBC and HT (accuracy: 74%, **95%CI = 0.64–0.90**) as the most promising to distinguish FEP-nt from HC; WBC and %Neu to allocate to FEP-t or HC group (accuracy: 87%, **95%CI = 0.64–0.90**); RDW-SD and LYMPH (accuracy: 86%, **95% CI = 0.75–97**) for distinguishing FEP-nt from FEP-t. CBC could be a promising, cheap tool to determine abnormalities related to schizophrenia. However, more studies with larger sample sizes are required.

## Introduction

A complete blood count (CBC) test or full blood examination (FBE) is the most commonly performed blood examination, which contributes to the evaluation of overall health. The method is simple, economical, and routinely carried out on all patients to monitor clinical changes during hospitalization or ambulatory treatment. CBC is often used in psychiatric patients to examine general health conditions or monitor changes in blood parameters related to pharmacotherapy [1].

CBC may reflect the severity of conditions with low-grade inflammation features different from those triggered by acute and chronic infections. Changes in the neutrophil-to-lymphocyte ratio (NLR) were connected with many chronic diseases, e.g. diabetes mellitus, hypertension, metabolic syndrome, obesity, lipid homeostasis disruption, ischemic stroke, and cerebral haemorrhage and are additional long-term prognostic biomarker [2, 3]. All these conditions have subclinical inflammatory mechanisms in common [4].

Schizophrenia-associated low-grade inflammation is a vital determinant of patient outcomes. Various markers of inflammation were tested over the years [5, 6]. Stratification based on inflammation graduation may help predict response to treatment and future prognosis [7]. Nevertheless, most of the proposed assays are not routinely available. Some blood morphology components could predict inflammatory processes, including total leukocytes, white blood count (WBC), and red cell distribution width. Available articles are heterogeneous, which hamper unambiguous conclusions [8–10].

NLR is a useful marker of inflammation in schizophrenia and other psychiatric disorders, including major depression, bipolar disorder, and individuals who attempted suicide [11–14]. Evermore frequently researchers indicate that NLR conveys pro-/anti-inflammatory homeostasis information in psychosis [15–17]. Other markers that reflect inflammatory processes are platelet-to-lymphocyte ratios (PLR) and monocyte-to-lymphocyte ratios (MLR). In bipolar disorder, PLR was linked with the onset and recurrence of hypomanic episodes [13]. MLR and PLR values in schizophrenia patients during the remission period were significantly higher than in healthy individuals, which supports the inflammation hypothesis of the disease [11].

Schizophrenia (SZ) and psychotic symptoms are related to other multisystem biological deregulations, including oxidative stress (OS). Increased vulnerability to OS was reported in individuals suffering from psychiatric disorders [18]. The pro/antioxidant status is a suggested predictor of schizophrenia risk and an indicator of symptom severity [19]. Patients during various stages of illness and treated by different classes of antipsychotics and other drugs could be characterized by heterogeneous pro/antioxidant alterations. Unfavourable oxidative changes are linked with worse clinical outcomes, more pronounced cognitive impairment and depressive symptoms, and poorer response to treatment [20, 21].

Despite the long history of performed CBC and open access to results of this examination in a large cohort of patients, only a few studies assess the importance of changes in CBC in the population of psychiatric patients [11, 15, 22, 23]. In schizophrenia, NLR was correlated with C Reactive Protein (CRP) and OS parameters, but the amount of evidence is still less confidently supportive of this conclusion [16, 24]. The aim of the study was to assess the utility of CBC in the prediction of schizophrenia diagnosis and the relationship between CBC and OS in the first-episode patient population.

## Materials and methods

### Study population

The study population included participants aged between 16 and 40 years: 47 individuals with the first acute episode of psychosis (FEP group): drug-naive (FEP-nt, n = 26), and under antipsychotic treatment (FEP-t, n = 21) and 30 healthy persons, as a control group (HC). The participants of the study were recruited: the FEP group from inpatients at the 1st Department of Psychiatry, Psychotherapy and Early Intervention of the Medical University of Lublin, Neuropsychiatric Hospital in Lublin and outpatients from private medical practice psychiatrists from Lublin, and the HC group—from the Department staff, their relatives and students. The recruitment period for the project lasted from February 2014 to November 2016. The inclusion and exclusion criteria for the FEP and HC groups are presented in Table 1.

**Table 1:** The inclusion and exclusion criteria for the study.

Inclusion criteria	Exclusion criteria
For the FEP and HC group	
1. written informed consent* obtained from study participants or/and their parents/legal guardians to: a) take part in the study; b) process of personal data (RODO-Polish Data Protection Law) as part of this project; 2. age between 16 to 40 years; 3. both sex;	1. lack of written informed consent* obtained from study participants or/and their parents/legal guardian to: a) take part in the study; b) process of personal data (RODO-Polish Data Protection Law) as part of this project; 2. any neurological diseases or organic brain dysfunction; 3. intellectual disability; 4. autoimmune diseases or other diseases in unstable phases (including metabolic disorders); 5. any addiction (except nicotine and caffeine); 8. the present clinical signs of inflammation (as in our previous study interpreted as hsCRP≧ 5 μg/ml) and/or leucocytosis (>10.000 G/I) during entry to the examination; 9. pregnancy and/or lactation in the case of women.
Additional criteria for:	
FEP group	HC group
4. meeting the criteria of schizophrenia according to the Diagnostic and Statistical Manual of Mental Disorders, Fifth Edition (DSM-5) [25], from 0 to 24 months after first treatment contact.	10. any psychotic diagnosis in the past or/and in the first-degree relatives.

(*according to Polish law, obtaining informed and voluntary consent depends on the age of the minors:

• below 16 years old—only parental/legal guardian consent is required,

•from 16 to 18 years of age—the consent of both parents and the minor is obligatory)

The study was conducted with the ethical principles of the Declaration of Helsinki [26]. The ethical approval was obtained from the Ethics Committee of the Medical University of Lublin, Poland (ID of the permission: KE-0254/231/2013) and all participants included in this study gave written informed consent.

### Data collection

#### Biochemical procedures

Venous blood samples (20 ml) were obtained after overnight fasting in Monovette blood collection tubes (S-Monovette ^®^ Serum). Next, samples were centrifuged, obtained serum was aliquoted and frozen at −80 °C until analysis. In a group of hospitalized patients, blood samples were collected within the first 24 hours of hospitalisation, in an outpatient group during the visit.

The following parameters were evaluated in obtained samples: white blood cells (WBC), red blood cells (RBC), haemoglobin (Hb), haematocrit (Ht), mean corpuscular value (MCV), mean cell haemoglobin (MCH), mean corpuscular haemoglobin concentration (MCHC), red cell distribution width (RDW), neutrophils (NEU), lymphocytes (LYMPH), monocytes (MONO), eosinophils (EOZ), basophils (BASO), platelets (PLT), platelets distribution width (PDW), mean platelet volume (MPV), platelets large cell ratio (P-LCR). Based on the obtained results, neutrophils to lymphocytes ratios (NLR), platelets to lymphocytes ratios (PLR), and monocyte to lymphocytes ratios (MLR) were also calculated. To analyse oxidative stress biomarkers, we use the protocol described in our earlier works [19, 27]. The pro/antioxidant balance assessment included enzymatic and non-enzymatic antioxidants (catalase (CAT), glutathione peroxidase (GPx), superoxide dismutase-1 (SOD-1), glutathione reductase (GR), reduced glutathione (GSH), total antioxidant capacity (TAC), and ferric reducing ability of plasma (FRAP)) and oxidative damage products (advanced glycation end products (AGEs), advanced oxidation protein products (AOPP), di-tyrosine (DITYR), kynurenine (KYN), N-formyl kynurenine (NFK), tryptophan (TRY), and total oxidant status (TOS), as well as concentrations of nitric oxide (NO)).

### Sociodemographic and clinical data

The clinical data was recorded by the supervising physician during the blood collection day.

#### Self-constructed questionnaire

Information about the study population group was obtained using a self-constructed questionnaire consisting of socio-demographic, lifestyle- and health-related questions. The questions concerning gender, age, body mass index (BMI), and medications were included. The participants filled out the questionnaire together with the researcher. The equivalents of antipsychotic medication were calculated based on defined daily doses (DDDs) presented by the World Health Organization’s Collaborative Centre for Drug Statistics Methodology and were presented to 1 mg olanzapine [28].

#### Positive and negative symptoms scale

A well-trained physician conducted the structured interview to assess the severity of schizophrenia symptoms in the patient group using the Positive and Negative Symptoms Scale (PANSS) in the Polish adaptation [29]. The assessment was performed on the day of blood collection and clinical examinations.

### Statistical analysis

Data analysis was conducted using the Statistica 13 Software (TIBCO Software Inc., Palo Alto, CA, USA). Firstly, the distribution of continuous variables was determined by the Shapiro-Test Wilk. Due to the non-Gaussian distribution, we used a nonparametric test for further analyses. Socio-demographic data were presented as median (M), interquartile range (IQR), or percentage of examined subgroup in the case of qualitative variables. To compare differences in examined factors between groups, we applied the chi-square test (qualitative variables), the U-Mann Whitney test (two subgroups), and the Kruskal-Wallis test with post-hoc analyses (three subgroups). We calculated the rho-Spearman rank correlation to assess the relationship between examined variables. The value of p < 0.05 was considered statistically significant. In the case of analysed data from more than two subgroups p-value was adjusted for multiple comparisons using the Bonferroni correction.

The classification tree model method (C&RT) was used to assess potential predictors helpful in recognizing FEP patients. C&RT is the most often used data mining method, which allows selecting more promising factors from many variables to categorize examined groups into characteristic subgroups (f.e., based on the presence or absence of specific diagnosis like schizophrenia). All chosen variables were categorized hierarchically and had a specific cut-off point that allowed allocation to subgroups. Examining factors are split into two subset trees during the classification according to cut-off points. Each of the following subsets maximizes the homogeneity of the two resulting subgroups [30, 31]. For C&RT results, we calculated the area under the receiver operating characteristic curve (AUC) for all models, which measures discrimination accuracy.

## Results

### Sociodemographic characteristics of the examined population

The characteristic of the examined group is depicted in Table 2. Half of the participants from FEP and one-third from the HC group were males. There were no differences between age (M: 21 years in FEP and M: 24 years in HC) and BMI (M: 21.3 kg/m^2^ in FES and 21.6 kg/m^2^ in HC) between healthy individuals and patients (p>0.05). As could be expected, we found differences between the doses of antipsychotic medication (M: 0 in FEP-nt and M: 8.75 in FEP-t; p<0.001) and the duration of illness (M: 1 month in FES-nt, M: 19 months in FES-t) in treated and drug-naive patients. The patients’ subgroup did not differ in the severity of schizophrenia symptoms and age of onset (p>0.05).

**Table 2:** Socio-demographic and clinical characteristics of the examined population.

Variable	Group	M	IQR	Differences between groups
Gender (n, % male)	FEP	25	53.19	p = 0.16
	HC	11	36.67	
Age (years)	FEP	21	5	p = 0.06
	HC	24	4	
BMI (kg/m^2^)	FEP	21.3	3.71	p = 0.85
	HC	21.6	5.3	
Age of onset	FEP-nt	21.5	6	p = 0.57
	FEP-t	20	5	
Duration of illness (months)	FEP-nt	1	2.25	p<0.001
	FEP-t	19	19	
Dose of olanzapine equivalents (mg)	FEP-nt	0	0	p<0.001
	FEP-t	8.75	13.75	
PANSS positive	FEP-nt	26	8	p = 0.22
	FEP-t	24	9	
PANSS negative	FEP-nt	21	11	p = 0.06
	FEP-t	25	15	
PANSS general	FEP-nt	52	13.5	p = 0.51
	FEP-t	54	9	
PANSS total	FEP-nt	101	21	p = 0.43
	FEP-t	107	29	

M–median, IQR—interquartile range; BMI–body mass index; HC–healthy controls; FEP–first-episode psychosis; ut–untreated (drug-naive); t–treated; PANSS–Positive and Negative Symptoms Scale

### The differences in CBC parameters between SZ and HC

The changes in CBC in the SZ population are presented in Table 3 and S1 Table. According to performed analysis, SZ had higher levels of WBC (p = 0.004), MCHC (p = 0.04), NEU (p = 0.04), MONO (p = 0.02), EOZ (p = 0.002), BASO (p = 0.03), and %EOZ (p = 0.01). Some studies suggest the effect of antipsychotic drugs on blood parameters [32]. To assess disease-related changes not dependent on treatment, we analysed subgroups: drug-naive (untreated: FEP-nt) and received antipsychotic medication (treated: FEP-t) patients. The results of these analyses are depicted in Table 4 and S2 Table. WBC levels were different between FEP-t and HC groups (p = 0.008), and LYMPH levels were higher in FEP-t than in both HC and FEP-nt (p = 0.01). PLR was higher in FEP-nt than in FEP-t (p = 0.025), and MONO was higher in FEP-t than in HC (p = 0.038). EOZ (p = 0.007) and BAZO (p = 0.043) were lower in HC than the FEP-t. Differences in PLR, MONO, and BASO were not significant after Bonferroni correction (p>0.017).

**Table 3:** Comparison of blood parameters between patients and healthy individuals.

Variable	Group	M	IQR	Differences between groups
WBC	FEP	6.65	2.65	p = 0.004
	HC	5.83	0.99	
MCHC	FEP	35.3	1.3	p = 0.04
	HC	34.8	1.2	
NEU	FEP	3.4	1.16	p = 0.04
	HC	2.9	1.08	
MONO	FEP	0.61	0.34	p = 0.02
	HC	0.51	0.11	
EOZ	FEP	0.21	0.18	p = 0.002
	HC	0.11	0.11	
BASO	FEP	0.04	0.03	p = 0.03
	HC	0.02	0.01	
%EOZ	FEP	3.15	2.5	p = 0.01
	HC	2	1.5	

M–median, IQR—interquartile range; HC–healthy controls; FEP–first-episode psychosis; WBC—white blood cells; MCHC—mean corpuscular haemoglobin concentration; NEU–neutrophils; MONO–monocytes; EOZ–eosinophils; BASO–basophils; %EOZ–eosinophils percentage

**Table 4:** Comparison of blood parameters between patients’ subgroups and healthy individuals.

Variable	Group	M	IQR	Differences between groups	Groups with differences
WBC	FEP-nt	6.09	3.22	p = 0.008	FEP-t>HC
	FEP-t	6.721	1.31		
	HC	5.83	0.99		
LYMPH	FEP-nt	1.9	0.7	p = 0.01	FET-ut<FEP-t FET-t>HC
	FEP-t	2.4	1.09		
	HC	2.1	0.5		
PLR	FEP-nt	133.55	67.36	p = 0.025	FEP-nt>-FEP-t
	FEP-t	96.65	36.55		
	HC	113.06	35.10		
MONO	FEP-nt	0.56	0.49	p = 0.038	FEP-t>HC
	FEP-t	0.62	0.26		
	HC	0.51	0.11		
EOZ	FEP-nt	0.18	0.16	p = 0.007	FEP-t>HC
	FEP-t	0.22	0.21		
	HC	0.11	0.11		
BASO	FEP-nt	0.04	0.03	p = 0.043	FEP-t>HC
	FEP-t	0.04	0.04		
	HC	0.02	0.01		

M–median, IQR—interquartile range; HC–healthy controls; FEP–first-episode psychosis; nt–untreated (drug-naive); t–treated; WBC—white blood cells; LYMPH–lymphocytes; MONO–monocytes; EOZ–eosinophils; BASO–basophils; PLR—platelets to lymphocytes ratio

### The relationship between clinical variables and CBC

The relationships between clinical variables and CBC are shown in Table 5. In HC there were many relationships between blood parameters and BMI (RBC: R = 0.63; Hb: R = 0.62, Ht: R = 0.58, MCHC: R = 0.55 and PLT: R = -0.46, p<0.05). In all patients with SZ, we found a correlation between the age at onset and MCV (R = 0.41, p<0.05) and MCHC (R = 0.44, p<0.05), duration of psychosis and MCHC (R = -0.40, p<0.05), and duration of illness and RDW-SD (R = 0.53, p<0.05). We did not find any correlation between clinical variables in CBC in the FEP-t group. In FEP-nt MCV and MCHC were related to age (R = 0.57, R = 0.49; p<0.05, respectively) and age at onset (R = 0.55, R = 0.47, p<0.05, respectively). MCHC was connected with the duration of psychosis (R = -0.44, p<0.05) and the symptoms of schizophrenia: positive (R = 0.44, p<0.05) and general (R = 0.46, p<0.05). RDW-SD was related to the duration of psychosis (R = 0.54, p<0.05) and the duration of illness (R = 0.52, p<0.05). Positive symptoms correlated with WBC (R = 0.54, p<0.05), and %BASO (R = 0.52, p<0.05), while general symptoms by RDW-CV (R = -0.49, p<0.05).

**Table 5:** Correlations between CBC and clinical variables.

**HC**					
**Variable** *	**RBC**	**Hb**	**Ht**	**MCHC**	**PLT**
**BMI**	0.63	0.62	0.58	0.55	-0.46

**FEP**				
**Variable** *	**MCV**	**MCHC**	**MCHC**	**RDW-SD**
**Age of onset**	0.41	0.44		
**Duration of psychosis**			-0.40	
**Duration of illness**				0.53

**FEP-nt**							
**Variable** *	**WBC**	**MCV**	**MCH**	**MCHC**	**RDW-SD**	**RDW-CV**	**%BASO**
**Age**		0.57	0.49				
**Age of onset**		0.55	0.47				
**Duration of psychosis**				-0.40	0.54		
**Duration of illness**					0.52		
**PANSS positive**	0.54			0.44			-0.52
**PANSS general**				0.46		-0.49	

HC–healthy controls; FEP–first-episode psychosis; nt–untreated (drug-naive); WBC—white blood cells; RBC—red blood cells; Hb—haemoglobin, Ht–haematocrit; MCV—mean corpuscular value; MCH—mean cell haemoglobin, MCHC—mean corpuscular haemoglobin concentration; RDW–red cell distribution width; RDW-SD–red cell distribution width–standard deviation, RDW-CV—red cell distribution width–coefficient of variation; %BASO–basophils percentage; PLT–platelets; PANSS—Positive and Negative Symptoms Scale

*only correlations upon 0.4 were shown

### The relationship between CBC and OS

We found various relationships between OS and CBC parameters, which are presented in Table 6. In HC SOD-1 was related to: WBC (R = -0.42, p<0.04), Hb (R = -0.41, p<0.05), MCHC (R = -0.51, p<0.05), RDW-SD (R = 0.58, p<0.05), RDW-CV (R = 0.52, p<0.05). GR was connected to EOZ (R = 0.40, p<0.05) and %EOZ (R = 0.47, p<0.05). GSH was also connected to EOZ (R = 0.49, p<0.05) and %EOZ (R = 0.50, p<0.05) and MCHC additionally (R = 0.40, p<0.05). In healthy individuals, OSI was associated with EOZ (R = -0.45, p<0.05), KYN, and NFK with LYMP (R = -0.44, R = = 0.42, p<0.05, respectively). In this group, we revealed a relationship between AOPP and: RBC (0.53, p<0.05), Ht (R = 0.46, p<0.05), and EOZ (0.40, p<0.05).

**Table 6:** Correlations between CBC and OS.

**HC**										
**Variable** *	**WBC**	**RBC**	**Hb**	**Ht**	**MCHC**	**RDW-SD**	**RDW-CV**	**LYMPH**	**EOZ**	**%EOZ**
**SOD-1**	-0.42		-0.41		-0.51	0.58	0.52			
**GR**									0.40	0.47
**GSH**					0.40				0.49	0.50
**OSI**									-0.45	
**AOPP**		0.53		0.46					0.40	
**KYN**								-0.44		
**NFK**								-0.42		
**FEP-nt**										
**Variable** *	**WBC**	**Hb**	**Ht**	**MCHC**	**NEU**	**EOZ**	**BASO**	**%BASO**	**PLT**	**PDW**
**CAT**	**0.62**				0.43					
**SOD-1**		-0.42	-0.41							
**GSH**							0.47			
**FRAP**					0.42			-0.47		
**AGEs**				0.46						
**AOPP**							0.43		0.42	
**DITYR**										0.44
**KYN**										0.41
**NFK**				0.42						
**NO**						0.45				

**FEP-t**								
**Variable** *	**Hb**	**Ht**	**NLR**	**PLR**	**MLR**	**%NEU**	**%MONO**	**PLT**
**CAT**								0.45
**GPx**			0.52			0.56		
**GR**	-0.47	-0.50						
**FRAP**			-0.51			-0.70		
**AOPP**					0.53		0.64	
**NO**				-0.54				

HC–healthy controls; FEP–first-episode psychosis; nt–untreated (drug-naive); t–treated; WBC—white blood cells; RBC—red blood cells; Hb—haemoglobin, Ht–haematocrit; MCHC—mean corpuscular haemoglobin concentration; RDW—red cell distribution width; RDW-SD–red cell distribution width–standard deviation, RDW-CV—red cell distribution width–coefficient of variation; NEU–neutrophils; %NEU–neutrophils percentage; LYMPH–lymphocytes; EOZ–eosinophils; %EOZ–eosinophils percentage; BASO–basophils; %BASO–basophils percentage; %MONO–monocytes; PLT–platelets; PDW- platelets distribution width; NLR—neutrophils to lymphocytes ratio; PLR—platelets to lymphocytes ratio; MLR–monocyte to lymphocytes ratio; CAT—catalase; GPx—glutathione peroxidase; SOD-1—superoxide dismutase-1; GR—glutathione reductase; GSH—reduced glutathione; OSI—oxidative stress index; FRAP—ferric reducing ability of plasma; AGEs—advanced glycation end products; AOPP—advanced oxidation protein products; DITYR—dityrosine; KYN—kynurenine; NFK—N-formyl kynurenine; NO—nitric oxide

*only correlations upon 0.4 were shown

In drug-naive patients there were relationships between CAT and: WBC (R = 0.62, p<0.05), NEU (R = 0.43, p<0.05); SOD-1 and: Hb (R = -0.42, p<0.05), Ht (R:-0.41, p<0.05); FRAP and: %BASO (R = -0.47, p<0.05), NEU (R = 0.42, p<0.05), AOPP and: BASO (R = 0.43, p<0.05), PLT (R = 0.42, p<0.05). We found a connection between GSH and BASO (R = 0.47, p<0.05), AGEs, NFRK and MCHC (R = 0.46, R = 0.42, p<0.05, respectively), DITYR, KYN and PDW (R = 0.44, R = 0.41, p<0.05, respectively), NO and EOZ (R = 0.45, p<0.05) in FEP-nt.

In FEP-t there were relationships between GPx and NLR, %NEU (R = 0.52, R = 0.56, p<0.05, respectively), GR and Hb, Ht (R = -0.47, R = -0.50, p<0.05, respectively), FRAP and NLR, %NEU (R = -0.51, R = -0.70, p<0.05, respectively). In this patient’s subgroup, CAT was related with PLT (R = 0.45, p<0.05), AOPP to %MONO, and MLR (R = 0.64, R = 0.53, p<0.05) and NO to PLR (-0.54, p<0.05).

### The results of C&RT analysis of the first episode of schizophrenia patients

**Fig 1:**
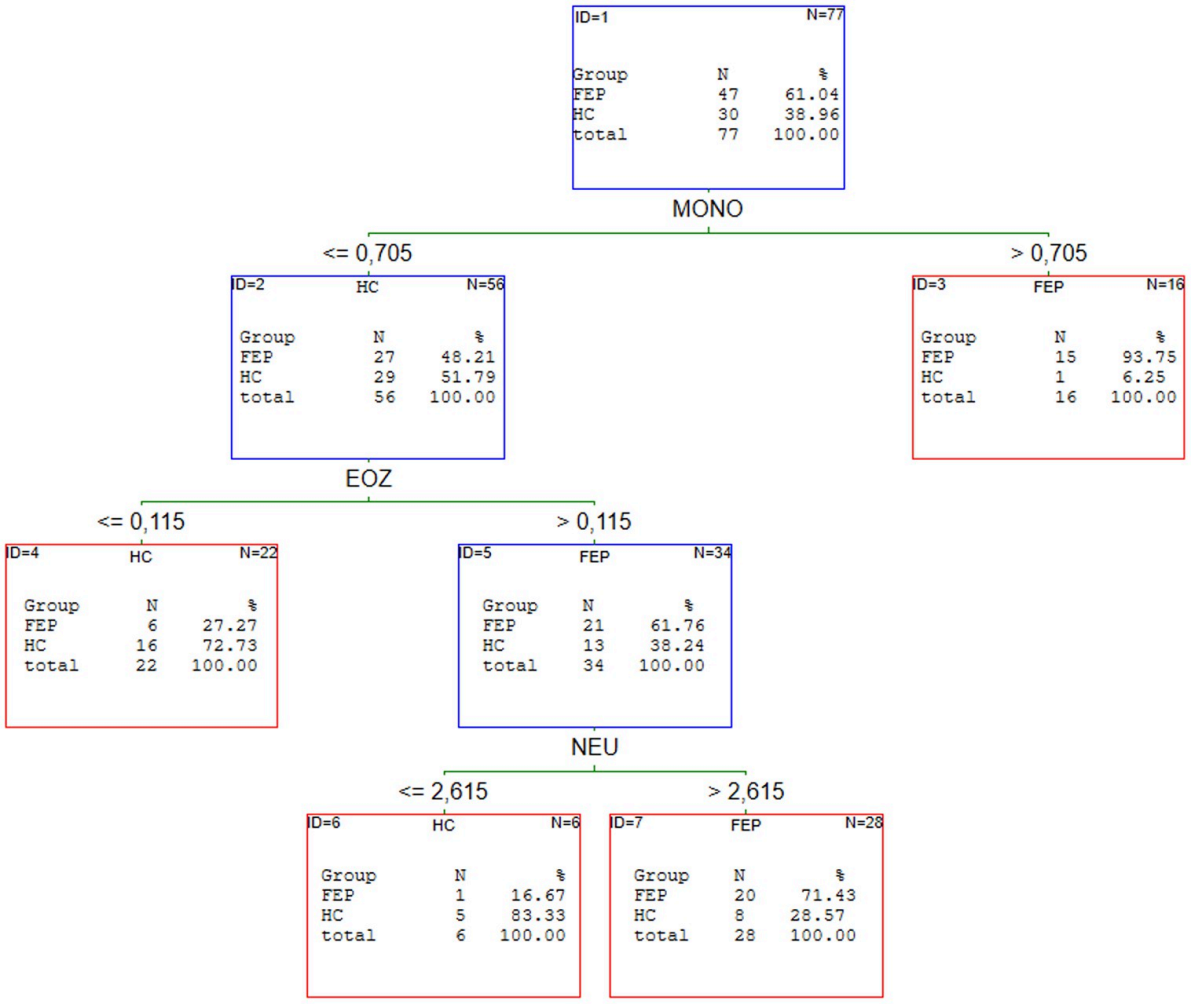
C&RT analyses for FEP and HC groups. HC–healthy controls; FEP–first episode psychosis; MONO–monocytes; EOZ–eosinophils; NEU–neutrophils.

**Fig 2:**
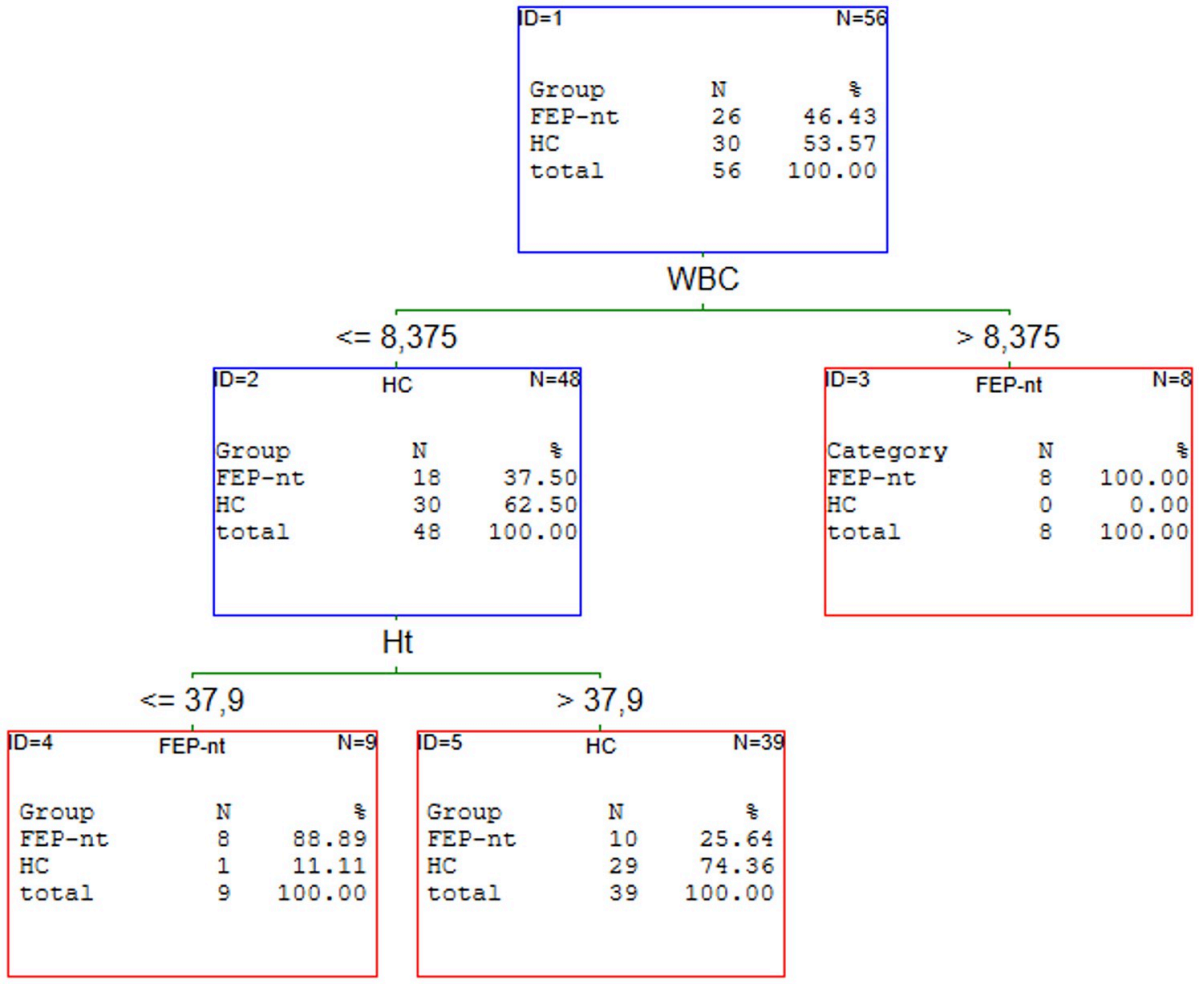
C&RT analyses for FEP-nt and HC groups. HC–healthy controls; FEP–first episode psychosis; nt—untreated (drug-naive); WBC—white blood cells; Ht–hematocrit.

**Fig 3:**
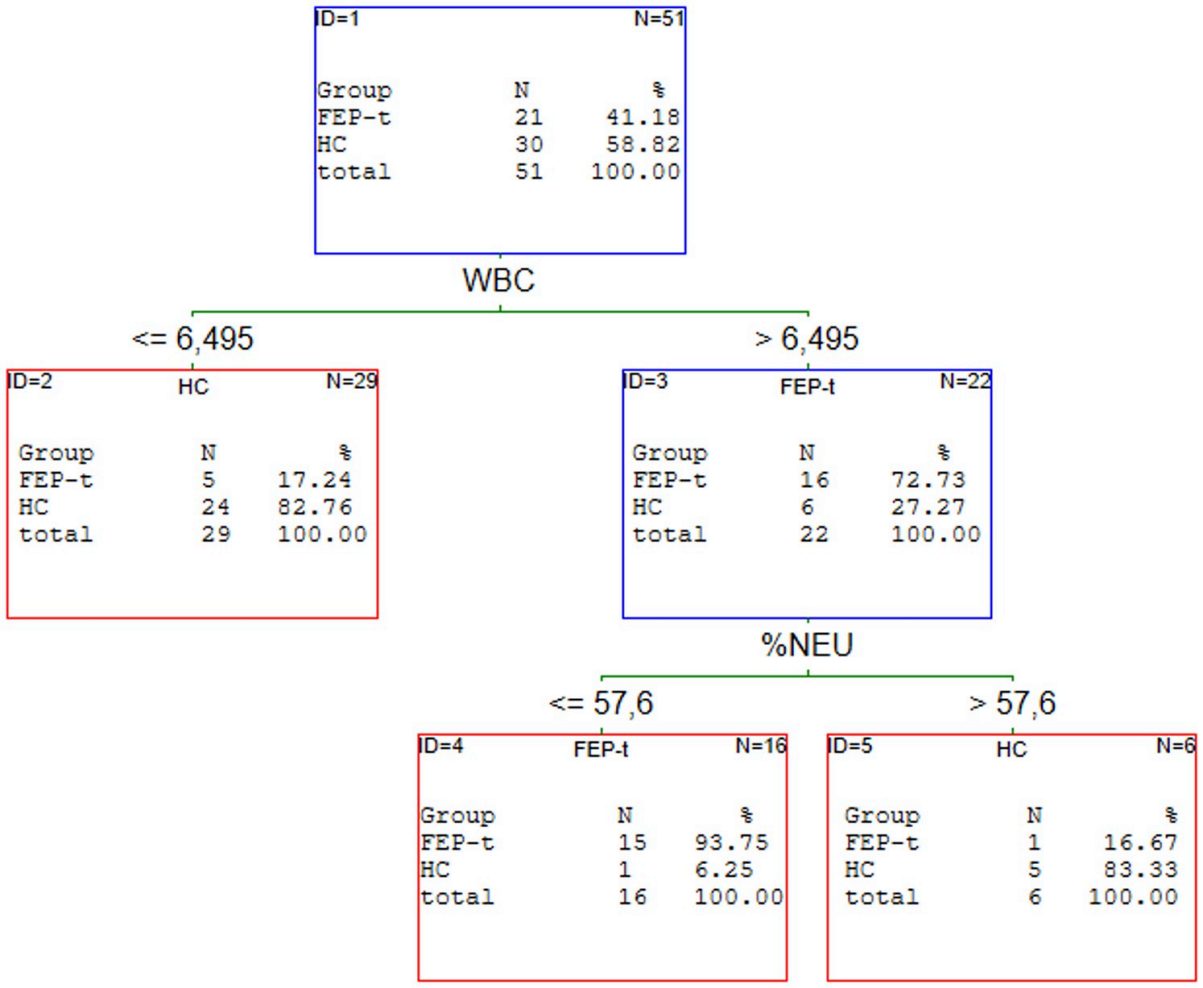
C&RT analyses for FEP-t and HC groups. HC–healthy controls; FEP–first episode psychosis; t–treated; WBC—white blood cells; %NEU–neutrophils (in %).

**Fig 4:**
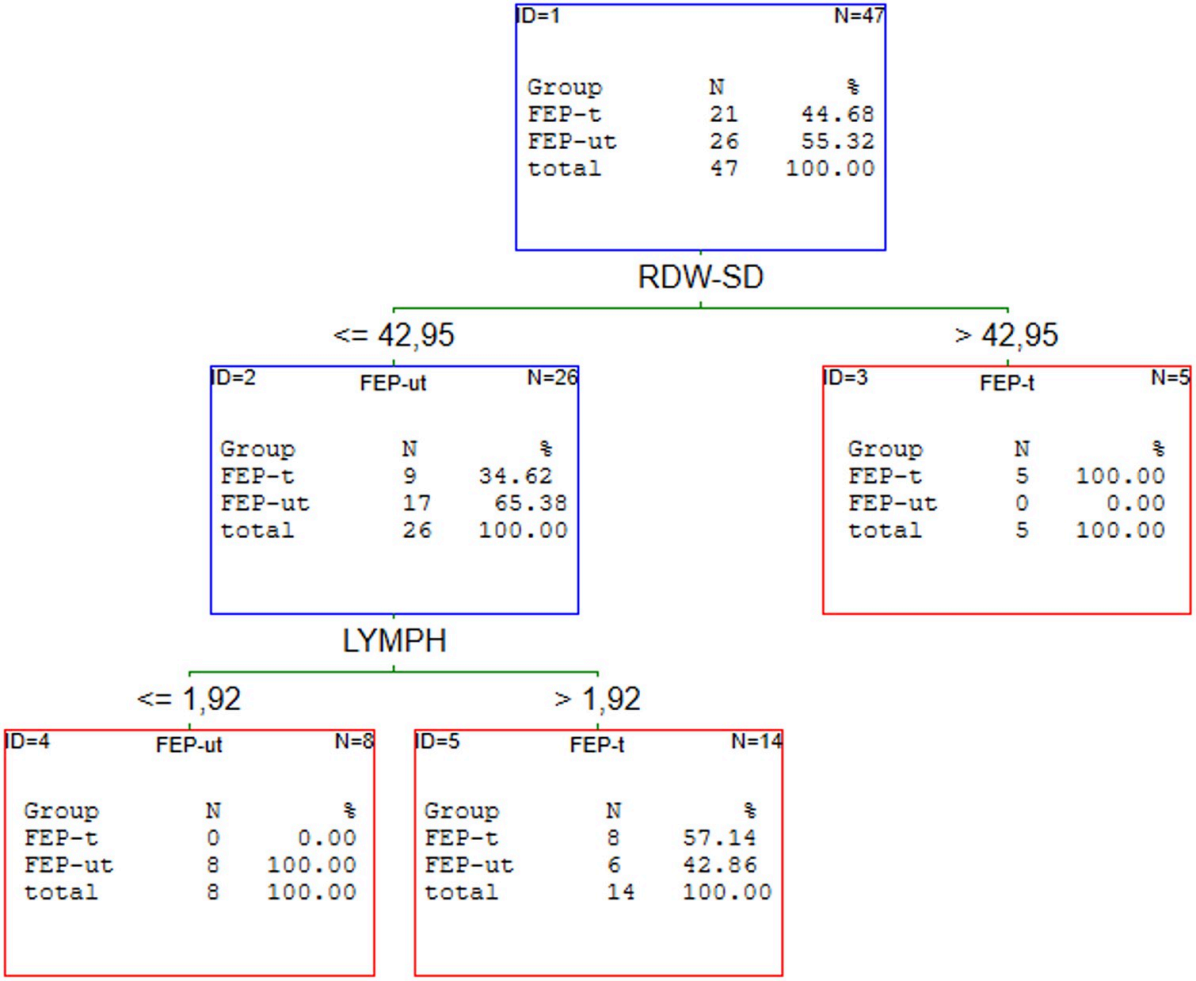
C&RT analyses for FEP-nt and FEP-t groups. FEP–first episode psychosis; t–treated; nt–untreated (drug-naive); LYMPH–lymphocytes; RDW-SD—red cell distribution width with standard deviation.

Our previous article proved that the redox state helps discriminate patients with schizophrenia from healthy individuals [19]. C&RT analysis was performed to find the potential panel of CBC markers most related to FEP. We analysed all FEP as a subgroup independent of treatment and two subgroups: drug-naive and received medication population. The results of the performed analyses are shown in Figs 1–4.

The most promising factors for discriminating FEP from HC were monocytes, eosinophils, and neutrophils with the cut-off points: >0.71 for MONO, >0.12 for EOZ, and >2.62 for NEU. The proposed model classifies the patients with 77% (**95%CI = 0.67–0.87)** accuracy. We found two factors to distinguish FEP-nt from HC: WBC with >8.40 cut-off point and HT with < = 37.9 cut-offs. The AUC value for the ROC curve was 74% (**95%CI = 0.64–0.90)**. The analysis singled out WBC and %NEU are most promising to allocate to the FEP-t or HC group. The cut-off point was: >6.50 for WBC and < = 57.6 for %NEU. This C&RT analysis achieved an accuracy of 87% (**95%CI = 0.76–0.99)**. Maximum accuracy for distinguishing the drug-naive first-episode patients from patients under treatment was attained with RDW-SD with a cut-off point <42.95 and LYMPH with a cut-off point < = 1.92. The proposed model divided treated and drug-naive patients with an accuracy of 86% (**95% CI = 0.75–97)**.

## Discussion

This study aimed to determine the potential utility of CBC in the prediction of the first episode of schizophrenia diagnosis and its relationship with OS in the patient population. WBC turned out to help distinguish both untreated and treated patients from healthy individuals, according to C&RT analysis. We found that FEP had higher WBC levels than HC (p = 0.004), significant only in the treatment population (p = 0.008). Nevertheless, in the drug-naive group, a higher WBC count was positively related to positive symptoms (R = 0.54; p<0.05).

In our study, a higher count of WBC was related to lower antioxidant defence represented by lower SOD-1 levels in the healthy group. In untreated patients, a higher level of WBC was related to more significant CAT activity. Considering potential relations between positive symptoms and WBC, it may be related to the increase in antioxidant activity as an adaptive mechanism to reduce schizophrenia symptoms [33]. This relationship was not revealed in the drug-received population what could be the effect of antipsychotic treatment on WBC. Leukocytes play a key role in the body’s defence mechanism. Multiple classes of WBC work together to maintain immune function, create antibodies, and participate in eliminating foreign materials [34]. Many reports confirm changes in WBC in SZ [11, 15, 32, 35]. Coordinators of immune-inflammatory responses (e.g. cytokines) provoke WBC differentiation [34].

The small sample size may result in a lack of differences in some CBC parameters after dividing the study population into treatment and drug-naive patients. In the meta-analysis, as mentioned above, patients under medication had WBC counts higher than healthy individuals [36]. Nevertheless, the authors did not assess drug-naive patients, which could give more opportunity to analyse and understand the relationship between antipsychotic drugs and blood immune parameters. Our study revealed WBC as the most promising CBC parameter in distinguishing FEP-nt from HC and HT with 74% accuracy. In another manuscript, WBC also was linked with schizophrenia symptoms. However, the relationship concerned negative symptoms [37]. It should be noted that higher WBC is a risk factor for metabolic syndrome and cardiovascular disease development in patients with non-affective psychosis. Heart disease is the leading cause of death in the SZ population. Added interpretation of CBC test results to the routine procedure of examination could more precisely predict cardiovascular risk in the patient’s group [35].

In our study, the FEP group also had higher MCHC compared to HC. These results were not dependent on whether or not treatment was received. MCHC correlates with the duration of illness; (R = -0.40, p<0.05), positive (R = 0.44, p<0.05), and general (R = 0.46, p<0.05) symptoms in drug-naïve population. MCHC is estimated by dividing HBG by Ht and expressing the average haemoglobin concentration per unit volume of red blood cells. Bentsen et al. found that MCHC was higher in SZ patients characterized by lower polyunsaturated fatty acids levels (PUFAs). This issue needs deeper insight in future papers, especially considering neuroprotective and anti-inflammatory PUFAs features and their relations with psychopathological symptoms [38–40].

NEU count was another variable that differentiated FEP and HC groups (p<0.04), and in the untreated population, a higher neutrophils count was linked to higher CAT activity and FRAP (R = 0.43, R = 0.42, p<0.05, respectively). Interestingly, in the FEP-t group, the percentage of NEU was inversely related to FRAP (R = -0.70, p<0.05) but also positively correlated to GPx activity (R = 0.56, p<0.05). Staying within the reference range of NEU before treatment was related to a better response to pharmacotherapy. Indeed, neutrophil count and NLR correlated significantly with positive symptoms of schizophrenia, and some authors suggest NLR as an indicator of two immune pathways may better predict an inflammatory state [41]. This was confirmed by meta-analysis, which indicated NLR and MLR were higher in SZ patients compared to healthy individuals [17]. In 2021, Dawidowski et al. proposed NLR as a tool for evaluating response to treatment in SZ [15]. Surprisingly, in our study, NEU was a better to define of FEP according to the C&RT model than NLR. Also, Nunez et al. proved the connection between NEU and SZ. After adjusting for several variables, NEU was related to brain tissue loss and more severe clinical symptomatology [42].

MONO was substantially higher in FEP than HC (p = 0.02), but analysis in subgroups revealed their count differentiated only from treated patients (p = 0.038). Among treated patients, the percentage of MONO may reflect AOPP levels (R = 0.64, p<0.05). Indeed, it has been shown that AOPP induces the oxidative metabolism of MONO [43]. Monocytes are sources of inflammatory biomolecules that trigger pro-inflammatory pathways. Our results are consistent with those obtained in other studies that indicated monocytosis as a characteristic future of acute SZ patients [17, 23, 44].

The median count of EOZ was approximately twice as high in the patients’ population than in the control group (p = 0.002), and this variable was different between treatment and healthy populations (p = 0.007). The levels of EOS were related to many OS markers: GR (R = 0.40, p<0.05), GSH (R = 0.49, p<0.05), OSI (R = -0.45, p<0.05), AOPP (R = 0.40, p<0.05) in healthy individuals, NO (R = 0.45, p<0.05) in drug-naïve patients. Eosinophilia is a non–dose-dependent effect of clozapine treatment [45]. In our study, seven patients received this medication, thus potentially affecting the obtained results. This phenomenon is confirmed by meta-analysis reveals no changes in the EOZ count in FEP [36]. We did not include this patient in the specific analysis due to the small size of the subgroup. Nevertheless, in our study, the EOZ was found to be a predictive factor of SZ diagnosis, with a cut-off point >0.12. Noteworthy, eosinophils are considered the cause of asthma via oxidative stress-dependent mechanisms. Overproduction of ROS could result from infiltrating immune cells [46]. The connections between EOZ and pro/antioxidant responses are also suggested in our results.

The median count of BASO was approximately twice as high in the patient population than in the control group (p = 0.03), and this variable was different between the treatment and healthy population (p = 0.043). It should be noted higher %BASO was inversely associated with positive symptoms (R = -0.52, p<0.05) and FRAP (R = -0.47, p<0.05) in FEP-nt. In the drug-naïve group, the count of BASO corresponds with GSH levels (R = 0.47, p<0.05) and AOPP (R = 0.43, p<0.05). Our results contradict the meta-analysis results from 2020 with no changes in BASO. Some suggest that the higher baseline basophils count is directly associated with a favourable outcome of SZ [47].

Our analysis suggests that antipsychotic medication could blur the results despite LYMPH differences between FEP and HC. According to subgroup analysis, FEP-t had a higher LYMPH count than HC (p = 0.01) and FEP-nt (p = 0.01). In healthy individuals, LYMPH corresponds with lower inflammatory marker levels: KYN (R = -0.44, p<0.05) and NFK (R = -0.42, p<0.05). Lymphocytes are the most studied CBC parameters in SZ. Mendelian randomization analyses suggest an association between higher lymphocyte counts and schizophrenia, which was not supported by a meta-analysis from 2020 [17, 48]. Nevertheless, some studies confirm the role of these parameters in acute SZ and their negative linkage with psychopathological symptoms [49]. The clinical utility of single-variable analysis could be unsatisfactory due to interaction networks between physiological processes. When we added RDW-SD to LYMPH in our research, the panel allowed distinguishing drug-naïve from treated patients with an 86% accuracy.

Also, PLR was different between drug-naïve patients (higher levels) and treated patients (p = 0.025). PLT are responsible for coordinating immune responses by regulating inflammatory molecules like, e.g., neutrophils and macrophages. Platelets affect the production of serotonin, and the PLT serotonin receptor is recognized as a peripheral marker of serotonin pathways [50]. In the FEP-nt group, PLR levels could suggest more severe oxidative stress reflected by AOPP (R = 0.42, p<0.05), but in FEP-t, higher activity of CAT—antioxidant enzymes (R = 0.45, p<0.05). The meta-analysis showed a trend of significance in PLT, with patients revealing higher FEP than healthy individuals. However, the authors did not perform analyses of these subgroups [17].

Significant antioxidants constitute a small fraction of the plasma thiol pool containing the–SH group. It has recently become more common to evaluate thiol/disulphide homeostasis as an indicator of oxidant/antioxidant balance [51]. The maintenance of thiol/disulphide balance is essential for the detoxification processes. Thiols are responsible for the non-enzymatic removal of reactive oxygen molecules via non-enzymatic pathways [52]. These parameters are proposed as novel oxidative stress parameters in numerous conditions, such as cardiovascular, neurological, skin, and respiratory diseases [53–56]. Little is known about thiol/disulphide balance in schizophrenia. However, it is demonstrated that shifted equilibrium toward the disulphide might be related to the pathogenesis of schizophrenia [51, 57]. This issue needs special attention in further research.

Some limitations of our analysis should be mentioned when interpreting the findings. Firstly, only selected OS biomarkers were assessed, and adding or changing some parameters may yield different results and conclusions. Many disruptions of the oxidative system exist in various chronic diseases and are not specific to psychiatric disorders. Numerous studies reported the effect of environmental and lifestyle factors on examined blood parameters. Also, using anti-inflammatory medications (corticosteroids, non-steroidal anti-inflammatory drugs) could interfere with the hemogram and OS parameters. Including these variables (e.g., diet, physical activity, tobacco use, air pollution) may strengthen the value of the obtained results. A relatively small study group was another limitation. An examination with a higher number of participants in the future is required.

## Conclusions

The study results confirm the changes in CBC blood tests in patients with first-episode schizophrenia and their relationship with clinical-related factors and oxidative stress biomarkers. Our analysis showed that the correlation between OS and CBC was significantly different between healthy individuals and patients–treated and drug-naïve. The probable explanation for this heterogeneity is the same schizophrenia process, which modulates antioxidant defence, and medication that modulates organism homeostasis. Nevertheless, in our study, many routinely assessed blood parameters could be a promising tool to determine abnormalities related to schizophrenia. However, more studies with higher sample sizes are required. The most promising models for discriminating: 1. FEP from HC was the combining of monocytes, eosinophils, and neutrophils (accuracy: 77%, **95%CI = 0.67–0.87**); 2. FEP-nt from HC was WBC and HT (accuracy: 74%, **95%CI = 0.64–0.90**); 3. FEP-t from HC was WBC and %Neu (accuracy: 87%, **95%CI = 0.76–0.99**); 4. FEP-nt from FEP-t was RDW-SD and LYMPH (accuracy: 86%, **95% CI = 0.75–97**). Considering the routine ordering CBC in patients with SZ as the initial blood test, the clinician and/or researcher should consider the possibility of interesting conclusions offered by more in-depth CBC results analysis. Further research in this field may be the first step towards implementing these results in clinical practice, taking into account assessing the accuracy of diagnosis or adherence with antipsychotic medication in schizophrenia.

## Supporting information

S1 Table Comparison of blood parameters between patients and healthy individuals. (PDF)

S2 Table Comparison of blood parameters between patients’ subgroups and healthy individuals. (PDF)

## References

[1] Virdee PS, Marian IR, Mansouri A, Elhussein L, Kirtley S, Holt T, et al. The Full Blood Count Blood Test for Colorectal Cancer Detection: A Systematic Review, Meta-Analysis, and Critical Appraisal. *Cancers (Basel)*. 2020; 12: E2348. doi: 10.3390/cancers12092348 32825191 PMC7564785

[2] Balta S, Ozturk C, Balta I, Demirkol S, Demir M, Celik T, et al. The Neutrophil-Lymphocyte Ratio and Inflammation. *Angiology*. 2016; 67: 298–299. doi: 10.1177/0003319715615252 26535014

[3] Song M, Graubard BI, Rabkin CS, Engels EA. Neutrophil-to-Lymphocyte Ratio and Mortality in the United States General Population. *Sci Rep*. 2021; 11: 464. doi: 10.1038/s41598-020-79431-7 33431958 PMC7801737

[4] Castro AM, Macedo-de la Concha LE, Pantoja-Meléndez CA. Low-Grade Inflammation and Its Relation to Obesity and Chronic Degenerative Diseases. *Revista Médica del Hospital General de México*. 2017; 80: 101–105. doi: 10.1016/j.hgmx.2016.06.011

[5] Fraguas D, Díaz-Caneja CM, Ayora M, Hernández-Álvarez F, Rodríguez-Quiroga A, Recio S, et al. Oxidative Stress and Inflammation in First-Episode Psychosis: A Systematic Review and Meta-Analysis. *Schizophr Bull*. 2019; 45: 742–751. doi: 10.1093/schbul/sby125 30169868 PMC6581144

[6] Mongan D, Ramesar M, Föcking M, Cannon M, Cotter D. Role of Inflammation in the Pathogenesis of Schizophrenia: A Review of the Evidence, Proposed Mechanisms and Implications for Treatment. *Early Interv Psychiatry*. 2020; 14: 385–397. doi: 10.1111/eip.12859 31368253

[7] Bishop JR, Zhang L, Lizano P. Inflammation Subtypes and Translating Inflammation-Related Genetic Findings in Schizophrenia and Related Psychoses: A Perspective on Pathways for Treatment Stratification and Novel Therapies. *Harv Rev Psychiatry*. 2022; 30: 59–70. doi: 10.1097/HRP.0000000000000321 34995036 PMC8746916

[8] Misiak B, Bartoli F, Carrà G, Stańczykiewicz B, Gładka A, Frydecka D, et al. Immune-Inflammatory Markers and Psychosis Risk: A Systematic Review and Meta-Analysis. *Psychoneuroendocrinology*. 2021; 127: 105200. doi: 10.1016/j.psyneuen.2021.105200 33740587

[9] Cheng Y-C, Huang N, Wu J, Li C, Ke L, Li M, et al. The Prognostic Significance of Inflammation-Associated Blood Cell Markers in Patients with Upper Tract Urothelial Carcinoma. *Ann Surg Oncol*. 2016; 23: 343–351. doi: 10.1245/s10434-015-4781-z 26242371

[10] Sun H, Yin C, Liu Q, Wang F, Yuan C. Clinical Significance of Routine Blood Test-Associated Inflammatory Index in Breast Cancer Patients. *Med Sci Monit*. 2017; 23: 5090–5095. doi: 10.12659/MSM.906709 29069071 PMC5667583

[11] Özdin S, Böke Ö. Neutrophil/Lymphocyte, Platelet/Lymphocyte and Monocyte/Lymphocyte Ratios in Different Stages of Schizophrenia. *Psychiatry Res*. 2019; 271: 131–135. doi: 10.1016/j.psychres.2018.11.043 30472508

[12] Aydin Sunbul E, Sunbul M, Yanartas O, Cengiz F, Bozbay M, Sari I, et al. Increased Neutrophil/Lymphocyte Ratio in Patients with Depression Is Correlated with the Severity of Depression and Cardiovascular Risk Factors. *Psychiatry Investig*. 2016; 13: 121–126. doi: 10.4306/pi.2016.13.1.121 26766954 PMC4701675

[13] Fusar-Poli L, Natale A, Amerio A, Cimpoesu P, Grimaldi Filioli P, Aguglia E, et al. Neutrophil-to-Lymphocyte, Platelet-to-Lymphocyte and Monocyte-to-Lymphocyte Ratio in Bipolar Disorder. *Brain Sci*. 2021; 11: 58. doi: 10.3390/brainsci11010058 33418881 PMC7825034

[14] Ivković M, Pantović-Stefanović M, Dunjić-Kostić B, Jurišić V, Lačković M, Totić-Poznanović S, et al. Neutrophil-to-Lymphocyte Ratio Predicting Suicide Risk in Euthymic Patients with Bipolar Disorder: Moderatory Effect of Family History. *Compr Psychiatry*. 2016; 66: 87–95. doi: 10.1016/j.comppsych.2016.01.005 26995241

[15] Dawidowski B, Grelecki G, Biłgorajski A, Podwalski P, Misiak B, Samochowiec J. Effect of Antipsychotic Treatment on Neutrophil-to-Lymphocyte Ratio during Hospitalization for Acute Psychosis in the Course of Schizophrenia-A Cross-Sectional Retrospective Study. *J Clin Med*. 2021; 11: 232. doi: 10.3390/jcm11010232 35011972 PMC8745875

[16] Kulaksizoglu B, Kulaksizoglu S. Relationship between Neutrophil/Lymphocyte Ratio with Oxidative Stress and Psychopathology in Patients with Schizophrenia. *Neuropsychiatr Dis Treat*. 2016; 12: 1999–2005. doi: 10.2147/NDT.S110484 27574431 PMC4991539

[17] Mazza MG, Lucchi S, Rossetti A, Clerici M. Neutrophil-Lymphocyte Ratio, Monocyte-Lymphocyte Ratio and Platelet-Lymphocyte Ratio in Non-Affective Psychosis: A Meta-Analysis and Systematic Review. *World J Biol Psychiatry*. 2020; 21: 326–338. doi: 10.1080/15622975.2019.1583371 30806142

[18] Caldiroli A, Capuzzi E, Barkin JL, Grassi S, Esposito CM, Auxilia AM, et al. Is There an Association between Inflammatory/Anti-Oxidant Markers and the Presence of Psychotic Symptoms or Severity of Illness in Mood and Psychotic Disorders? A Multi-Centric Study on a Drug-Free Sample. *Brain Behav Immun Health*. 2022; 22: 100453. doi: 10.1016/j.bbih.2022.100453 35403068 PMC8990055

[19] Juchnowicz D, Dzikowski M, Rog J, Waszkiewicz N, Karakuła KH, Zalewska A, et al. Pro/Antioxidant State as a Potential Biomarker of Schizophrenia. *J Clin Med*. 2021; 10: 4156. doi: 10.3390/jcm10184156 34575267 PMC8466193

[20] Wei C, Sun Y, Chen N, Chen S, Xiu M, Zhang X. Interaction of Oxidative Stress and BDNF on Executive Dysfunction in Patients with Chronic Schizophrenia. *Psychoneuroendocrinology*. 2020; 111: 104473. doi: 10.1016/j.psyneuen.2019.104473 31655452

[21] Anderson G, Maes M, Berk M. Schizophrenia Is Primed for an Increased Expression of Depression through Activation of Immuno-Inflammatory, Oxidative and Nitrosative Stress, and Tryptophan Catabolite Pathways. *Prog Neuropsychopharmacol Biol Psychiatry*. 2013; 42: 101–114. doi: 10.1016/j.pnpbp.2012.07.016 22930036

[22] Zulfic Z, Weickert CS, Weickert TW, Liu D, Myles N, Galletly C. Neutrophil-Lymphocyte Ratio—a Simple, Accessible Measure of Inflammation, Morbidity and Prognosis in Psychiatric Disorders? *Australas Psychiatry*. 2020; 28: 454–458. doi: 10.1177/1039856220908172 32174125

[23] Sahpolat M, Ayar D, Ari M, Karaman MA. Elevated Monocyte to High-Density Lipoprotein Ratios as an Inflammation Markers for Schizophrenia Patients. *Clin Psychopharmacol Neurosci*. 2021; 19: 112–116. doi: 10.9758/cpn.2021.19.1.112 33508794 PMC7851456

[24] Jacomb I, Stanton C, Vasudevan R, Powell H, O’Donnell M, Lenroot R, et al. C-Reactive Protein: Higher During Acute Psychotic Episodes and Related to Cortical Thickness in Schizophrenia and Healthy Controls. *Front Immunol*. 2018; 9: 2230. doi: 10.3389/fimmu.2018.02230 30364161 PMC6192380

[25] *Diagnostic and Statistical Manual of Mental Disorders*: *DSM-5*; American Psychiatric Association, American Psychiatric Association, Eds.; 5th ed.; American Psychiatric Association: Washington, D.C. 2013. ISBN 978-0-89042-554-1.

[26] World Medical Association World Medical Association Declaration of Helsinki: Ethical Principles for Medical Research Involving Human Subjects. *JAMA*. 2013; 310: 2191–2194. doi: 10.1001/jama.2013.281053 24141714

[27] Juchnowicz D, Dzikowski M, Rog J, Waszkiewicz N, Zalewska A, Maciejczyk M, et al. Oxidative Stress Biomarkers as a Predictor of Stage Illness and Clinical Course of Schizophrenia. *Front Psychiatry* 2021, 12, 728986, doi: 10.3389/fpsyt.2021.728986 34867519 PMC8636114

[28] Leucht S, Samara M, Heres S, Davis JM. Dose Equivalents for Antipsychotic Drugs: The DDD Method. *Schizophr Bull*. 2016; 42 Suppl 1: S90–94. doi: 10.1093/schbul/sbv167 27460622 PMC4960429

[29] Kay SR, Fiszbein A, Opler LA. The Positive and Negative Syndrome Scale (PANSS) for Schizophrenia. *Schizophr Bull*. 1987; 13: 261–276. doi: 10.1093/schbul/13.2.261 3616518

[30] Muller R, Möckel M. Logistic Regression and CART in the Analysis of Multimarker Studies. *Clin Chim Acta*. 2008; 394: 1–6. doi: 10.1016/j.cca.2008.04.007 18455512

[31] Lewis RJ. An Introduction to Classification and Regression Tree (CART) Analysis. 14.

[32] Labonté C, Zhand N, Park A, Harvey PD. Complete Blood Count Inflammatory Markers in Treatment-Resistant Schizophrenia: Evidence of Association between Treatment Responsiveness and Levels of Inflammation. *Psychiatry Res*. 2022; 308: 114382. doi: 10.1016/j.psychres.2021.114382 34995832

[33] Murray AJ, Rogers JC, Katshu MZUH, Liddle PF, Upthegrove R. Oxidative Stress and the Pathophysiology and Symptom Profile of Schizophrenia Spectrum Disorders. *Front Psychiatry*. 2021; 12: 703452. doi: 10.3389/fpsyt.2021.703452 34366935 PMC8339376

[34] Abdulkhaleq LA, Assi MA, Abdullah R, Zamri-Saad M, Taufiq-Yap YH, Hezmee MNM. The Crucial Roles of Inflammatory Mediators in Inflammation: A Review. *Vet World*. 2018; 11: 627–635. doi: 10.14202/vetworld.2018.627-635 29915501 PMC5993766

[35] Moody G, Miller BJ. Total and Differential White Blood Cell Counts and Hemodynamic Parameters in First-Episode Psychosis. *Psychiatry Res*. 2018; 260: 307–312. doi: 10.1016/j.psychres.2017.11.086 29223800

[36] Jackson AJ, Miller BJ. Meta-Analysis of Total and Differential White Blood Cell Counts in Schizophrenia. *Acta Psychiatrica Scandinavica*. 2020; 142: 18–26. doi: 10.1111/acps.13140 31850530

[37] Fan X, Liu EY, Freudenreich O, Park JH, Liu D, Wang J, et al. Higher White Blood Cell Counts Are Associated with an Increased Risk for Metabolic Syndrome and More Severe Psychopathology in Non-Diabetic Patients with Schizophrenia. *Schizophr Res*. 2010; 118: 211–217. doi: 10.1016/j.schres.2010.02.1028 20189773

[38] Bentsen H, Solberg DK, Refsum H, Bøhmer T. Clinical and Biochemical Validation of Two Endophenotypes of Schizophrenia Defined by Levels of Polyunsaturated Fatty Acids in Red Blood Cells. *Prostaglandins Leukot Essent Fatty Acids*. 2012; 87: 35–41. doi: 10.1016/j.plefa.2012.05.005 22705264

[39] Goh KK, Chen A, Chen H, Lu ML. Effects of Omega-3 Polyunsaturated Fatty Acids Supplements on Psychopathology and Metabolic Parameters in Schizophrenia: A Meta-Analysis of Randomized Controlled Trials. *J Psychopharmacol*. 2021; 35: 221–235. doi: 10.1177/0269881120981392 33586517

[40] Li N, Yang P, Tang M, Liu Y, Guo W, Lang B, et al. Reduced Erythrocyte Membrane Polyunsaturated Fatty Acid Levels Indicate Diminished Treatment Response in Patients with Multi- versus First-Episode Schizophrenia. *Schizophr*. 2022; 8: 1–12. doi: 10.1038/s41537-022-00214-2 35217671 PMC8881498

[41] Sandberg AA, Steen VM, Torsvik A. Is Elevated Neutrophil Count and Neutrophil-to-Lymphocyte Ratio a Cause or Consequence of Schizophrenia?—A Scoping Review. *Front Psychiatry*. 2021; 12: 728990. doi: 10.3389/fpsyt.2021.728990 34603107 PMC8483576

[42] Núñez C, Stephan-Otto C, Usall J, Bioque M, Lobo A, González-Pinto A, et al. Neutrophil Count Is Associated With Reduced Gray Matter and Enlarged Ventricles in First-Episode Psychosis. *Schizophrenia Bulletin*. 2019; 45: 846–858. doi: 10.1093/schbul/sby113 30107610 PMC6581126

[43] Witko-Sarsat V, Gausson V, Nguyen T, Touam M, Drüeke T, Santangelo F, et al. AOPP-Induced Activation of Human Neutrophil and Monocyte Oxidative Metabolism: A Potential Target for N-Acetylcysteine Treatment in Dialysis Patients. *Kidney Int*. 2003; 64: 82–91. doi: 10.1046/j.1523-1755.2003.00044.x 12787398

[44] Drexhage RC, Hoogenboezem TA, Cohen D, Versnel MA, Nolen WA, van Beveren NJM, et al. An Activated Set Point of T-Cell and Monocyte Inflammatory Networks in Recent-Onset Schizophrenia Patients Involves Both pro- and Anti-Inflammatory Forces. *Int J Neuropsychopharmacol*. 2011; 14: 746–755. doi: 10.1017/S1461145710001653 21255481

[45] Lam YWF. Eosinophilia Associated with Clozapine Therapy. *The Brown University Psychopharmacology Update*. 2021; 32: 2–2. doi: 10.1002/pu.30740

[46] Michaeloudes C, Abubakar-Waziri H, Lakhdar R, Raby K, Dixey P, Adcock IM, et al. Molecular Mechanisms of Oxidative Stress in Asthma. *Mol Aspects Med*. 2022; 85; 101026. doi: 10.1016/j.mam.2021.101026 34625291

[47] Osimo EF, Perry BI, Cardinal RN, Lynall E, Lewis J, Kudchadkar A, et al. Inflammatory and Cardiometabolic Markers at Presentation with First-episode Psychosis and Long-Term Clinical Outcomes: A Longitudinal Study Using Electronic Health Records. *Brain Behav Immun*. 2021; 91: 117–127. doi: 10.1016/j.bbi.2020.09.011 32950620 PMC7773969

[48] Astle WJ, Elding H, Jiang T, Allen D, Ruklisa D, Mann AL, et al. The Allelic Landscape of Human Blood Cell Trait Variation and Links to Common Complex Disease. *Cell*. 2016; 167: 1415–1429.e19. doi: 10.1016/j.cell.2016.10.042 27863252 PMC5300907

[49] Nikkilä HV, Müller K, Ahokas A, Rimón R, Andersson LC. Increased Frequency of Activated Lymphocytes in the Cerebrospinal Fluid of Patients with Acute Schizophrenia. *Schizophr Res*. 2001; 49: 99–105. doi: 10.1016/s0920-9964(99)00218-2 11343869

[50] Aguglia A, Amerio A, Asaro P, Caprino M, Conigliaro C, Giacomini G, et al. High-Lethality of Suicide Attempts Associated with Platelet to Lymphocyte Ratio and Mean Platelet Volume in Psychiatric Inpatient Setting. *World J Biol Psychiatry*. 2021; 22: 119–127. doi: 10.1080/15622975.2020.1761033 32338121

[51] Ünal K, Erzin G, Yüksel RN, Alisik M, Erel Ö. Thiol/Disulphide Homeostasis in Schizophrenia Patients with Positive Symptoms. *Nordic Journal of Psychiatry*. 2018; 72: 281–284. doi: 10.1080/08039488.2018.1441906 29519188

[52] Erel O, Neselioglu S. A Novel and Automated Assay for Thiol/Disulphide Homeostasis. *Clinical Biochemistry*. 2014; 47: 326–332. doi: 10.1016/j.clinbiochem.2014.09.026 25304913

[53] Caliskan HM, Sivri S, Sokmen E, Celik M, Ilanbey B, Ozbek SC, et al. Prognostic Value of Thiol/Disulfide Homeostasis in Symptomatic Patients with Heart Failure. *Arch Physiol Biochem*. 2021; 127: 462–467. doi: 10.1080/13813455.2020.1773505 32497442

[54] Kocatürk M, Kirmit A. Evaluation of IL-10, IFN-γ, and Thiol–Disulfide Homeostasis in Patients with Drug-Resistant Epilepsy. *Neurol Sci*. 2022; 43: 485–492. doi: 10.1007/s10072-021-05331-x 34036451

[55] Ozyurt S, Ozcelik N, Kara BY, Arpa M, Metin Y, Erel O, et al. Evaluation of Thiol/Disulfide Homeostasis in Bronchiectasis. *Canadian Respiratory Journal*. 2022; 2022: e8340450. doi: 10.1155/2022/8340450 35132344 PMC8817889

[56] Georgescu SR, Mitran CI, Mitran MI, Matei C, Popa GL, Erel O, et al. Thiol-Disulfide Homeostasis in Skin Diseases. *Journal of Clinical Medicine*. 2022; 11: 1507. doi: 10.3390/jcm11061507 35329832 PMC8954849

[57] Topcuoglu C, Bakirhan A, Yilmaz FM, Neselioglu S, Erel O, Sahiner SY. Thiol/Disulfide Homeostasis in Untreated Schizophrenia Patients. *Psychiatry Research*. 2017; 251: 212–216. doi: 10.1016/j.psychres.2017.02.016 28214778

