# Supplementary material for: The usefulness of a complete blood count in the prediction of the first episode of schizophrenia diagnosis and its relationship with oxidative stress
Source: PLoS One. 2023 Oct 12;18(10):e0292756. doi: 10.1371/journal.pone.0292756 (PMC10569548; doi:10.1371/journal.pone.0292756)
Supplement: S1 Table — (PDF) [file pone.0292756.s001.pdf]

S1 Table. Comparison of blood parameters between patients and healthy individuals.

Variable	Group	M	IQR	Differences between groups
WBC*	FEP	6.65	2.65	p=0.004
	HC	5.83	0.99	
RBC	FEP	4.81	0.61	p=0.94
	HC	4.82	0.44	
Hb	FEP	14.5	2	p=0.99
	HC	14.3	1.7	
Ht	FEP	40.9	5	p=0.59
	HC	41.5	4.4	
MCV	FEP	85.4	4.9	p=0.26
	HC	86.2	4.7	
MCH	FEP	30.1	1.8	p=0.91
	HC	30.15	1.5	
MCHC*	FEP	35.3	1.3	p=0.04
	HC	34.8	1.2	
RDW-SD	FEP	39.3	3.6	p=0.78
	HC	39.4	3.3	
RDW-CV	FEP	12.6	0.9	p=0.70
	HC	13	1	
NEU*	FEP	3.4	1.16	p=0.04
	HC	2.9	1.08	
LYMPH	FEP	2.19	0.81	p=0.37
	HC	2.1	0.5	
NLR	FEP	1.61	0.56	p=0.30
	HC	1.37	0.77	
PLR	FEP	118.5	46.81	p=0.85
	HC	113.06	35.10	

MLR	FEP	0.26	0.10	p=0.34
	HC	0.25	0.12	
MONO*	FEP	0.61	0.34	p=0.02
	HC	0.51	0.11	
EOZ*	FEP	0.21	0.18	p=0.002
	HC	0.11	0.11	
BASO*	FEP	0.04	0.03	p=0.03
	HC	0.02	0.01	
%NEU	FEP	53.1	10.7	p=0.60
	HC	50.35	11.6	
%LYMPH	FEP	33.05	8.1	p=0.19
	HC	36.5	10.7	
%MONO	FEP	8.55	2.8	p=0.61
	HC	8.5	2.4	
%EOZ*	FEP	3.15	2.5	p=0.01
	HC	2	1.5	
%BASO	FEP	0.55	0.5	p=0.17
	HC	0.4	0.3	
PLT	FEP	249	86	p=0.86
	HC	247	52	
PDW	FEP	12.4	2.3	p=0.74
	HC	12.8	2.7	
MPV	FEP	10.6	1.2	p=0.68
	HC	10.6	1.3	
P-LCR	FEP	30.2	10.1	p=0.36
	HC	29.7	8.75	

M – median, IQR - interquartile range; HC – healthy controls; FEP – first episode psychosis; WBC - white blood cells; RBC - red blood cells; Hb - hemoglobin, Ht – hematocrit; MCV - mean corpuscular value; MCH - mean cell hemoglobin, MCHC - mean corpuscular hemoglobin concentration; RDW red cell distribution width; NEU – neutrophils; LYMPH – lymphocytes; MON – monocytes; EOZ – eosinophils; BASO – basophils; PLT – platelets; PDW- platelets distribution width; MPV - mean platelet volume; P-LCR - platelets large cell ratio; NLR - neutrophils to lymphocytes ratio; PLR - platelets to lymphocytes ratio; MLR – monocytes to lymphocytes ratio

*only correlations upon 0.4 were shown
