# Supplementary material for: The usefulness of a complete blood count in the prediction of the first episode of schizophrenia diagnosis and its relationship with oxidative stress
Source: PLoS One. 2023 Oct 12;18(10):e0292756. doi: 10.1371/journal.pone.0292756 (PMC10569548; doi:10.1371/journal.pone.0292756)
Supplement: S2 Table — (PDF) [file pone.0292756.s002.pdf]

S2 Table. Comparison of blood parameters between patients' subgroups and healthy individuals.

Variable	Group	M	IQR	Differences between groups	Groups with differences
	FEP-nt	6.09	3.22		
WBC*	FEP-t	6.721	1.31	p=0.008	FEP-t>HC
	HC	5.83	0.99		
	FEP-nt	4.89	0.63		
RBC	FEP-t	4.94	0.55	p=0.2	NA
	HC	4.82	0.44		
	FEP-nt	14.1	1.7		
Hb	FEP-t	14.9	1.5	p=0.25	NA
	HC	14.3	1.7		
	FEP-nt	39.75	4.8		
Ht	FEP-t	42.2	4.2	p=0.22	NA
	HC	41.5	4.4		
	FEP-nt	85.2	3.9		
MCV	FEP-t	85.5	5.4	p=0.5	NA
	HC	86.2	4.7		
	FEP-nt	30.15	1.7		
MCH	FEP-t	30	1.9	p=0.92	NA
	HC	30.15	1.5		
	FEP-nt	35.2	1.2		
MCHC	FEP-t	35.3	1.2	p=0.11	NA
	HC	34.8	1.2		
	FEP-nt	38.6	2.6		
RDW-SD	FEP-t	40.65	5.9	p=0.23	NA
	HC	39.4	3.3		
	FEP-nt	12.6	0.8		
RDW-CV	FEP-t	13.05	1.25	p=0.8	NA
	HC	13	1		
	FEP-nt	3.19	2.12		
NEU	FEP-t	3.7	0.97	p=0.81	NA
	HC	2.9	1.08		
LYMPH*	FEP-nt	1.9	0.7	p=0.01	FET-ut<FEP-t<br>FET-t>HC
	FEP-t	2.4	1.09		

	HC	2.1	0.5		
NLR	FEP-nt	1.73	0.75	p=0.18	NA
	FEP-t	1.52	0.46		
	HC	1.37	0.77		
PLR*	FEP-nt	133.55	67.36	p=0.025	FEP-nt>-FEP-t
	FEP-t	96.65	36.55		
	HC	113.06	35.10		
MLR	FEP-nt	0.28	0.18	p=0.25	NA
	FEP-t	0.24	0.09		
	HC	0.25	0.12		
MONO*	FEP-nt	0.56	0.49	p=0.038	FEP-t>HC
	FEP-t	0.62	0.26		
	HC	0.51	0.11		
EOZ*	FEP-nt	0.18	0.16	p=0.007	FEP-t>HC
	FEP-t	0.22	0.21		
	HC	0.11	0.11		
BASO*	FEP-nt	0.04	0.03	p=0.043	FEP-t>HC
	FEP-t	0.04	0.04		
	HC	0.02	0.01		
%NEU	FEP-nt	55.9	15.5	p=0.32	NA
	FEP-t	51	8.2		
	HC	50.35	11.6		
%LYMPH	FEP-nt	32	7.2	p=0.16	NA
	FEP-t	34.4	7.4		
	HC	36.5	10.7		
%MONO	FEP-nt	8.6	4.9	p=0.84	NA
	FEP-t	8.2	1.8		
	HC	8.5	2.4		
%EOZ	FEP-nt	3.2	2.4	p=0.037	NA
	FEP-t	3	3.3		
	HC	2	1.5		
%BASO	FEP-nt	0.6	0.4	p=0.37	NA
	FEP-t	0.4	0.5		
	HC	0.4	0.3		
PLT	FEP-nt	255.5	92	p=0.88	NA

	FEP-t	248	72		
	HC	247	52		
PDW	FEP-nt	12.2	1.4	p=0.54	NA
	FEP-t	13.45	2.3		
	HC	12.8	2.7		
MPV	FEP-nt	10.5	1.2	p=0.78	NA
	FEP-t	10.9	1.4		
	HC	10.6	1.3		
P-LCR	FEP-nt	29.7	9.9	p=0.51	NA
	FEP-t	32.85	11.55		
	HC	29.7	8.75		

M – median, IQR - interquartile range; HC – healthy controls; FEP – first episode psychosis; nt – untreated (drug-naive); t – treated; NA – not applicable; WBC - white blood cells; RBC - red blood cells; Hb - hemoglobin, Ht – hematocrit; MCV - mean corpuscular value; MCH - mean cell hemoglobin, MCHC - mean corpuscular hemoglobin concentration; RDW red cell distribution width; NEU – neutrophils; LYMPH – lymphocytes; MON – monocytes; EOZ – eosinophils; BASO – basophils; PLT – platelets; PDW- platelets distribution width; MPV - mean platelet volume; P-LCR - platelets large cell ratio; NLR - neutrophils to lymphocytes ratio; PLR - platelets to lymphocytes ratio; MLR – monocyte to lymphocytes ratio

*only correlations upon 0.4 were shown
